# Supplementary material for: FANCM promotes class I interfering crossovers and suppresses class II non-interfering crossovers in wheat meiosis
Source: Nat Commun. 2022 Jun 25;13:3644. doi: 10.1038/s41467-022-31438-6 (PMC9233680; doi:10.1038/s41467-022-31438-6)
Supplement: Supplementary file 1 — Supplementary Information [file 41467_2022_31438_MOESM1_ESM.pdf]

FANCM promotes class I interfering crossovers and suppresses class II non-interfering crossovers in wheat meiosis

Desjardins *et al.*

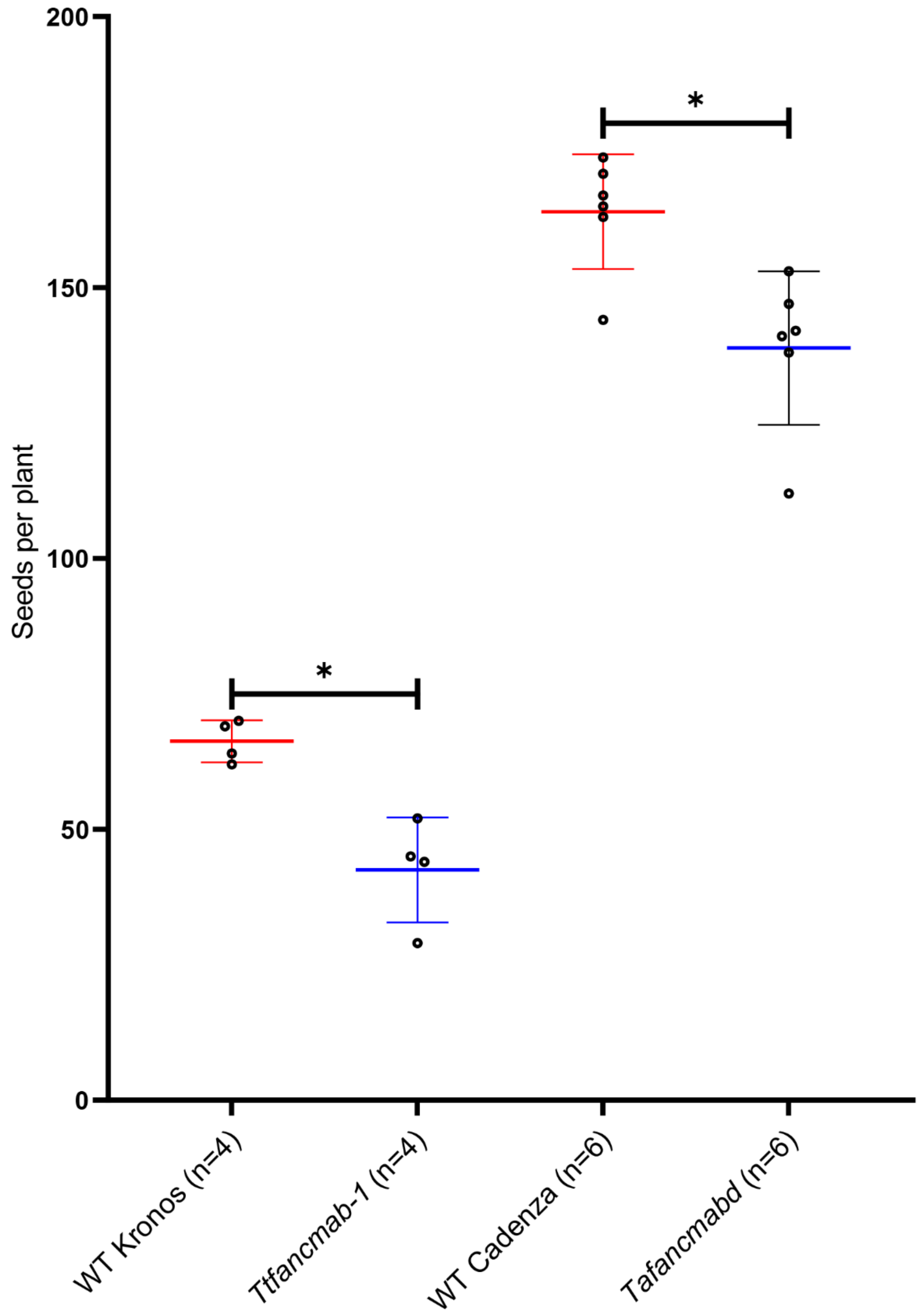

Supplementary Figure 1. Reduced seed set in *fancm* null mutants. Counts of total seed per plant with mean values ± SD. The number of plants sampled for each line is shown in brackets. * = $p < 0.05$ (Mann-Whitney U Test). Exact *p*-values are as follows: 1 vs 2 ($p = 0.03$) and 3 vs 4 ($p = 0.013$). Source data are provided as a Source Data file.

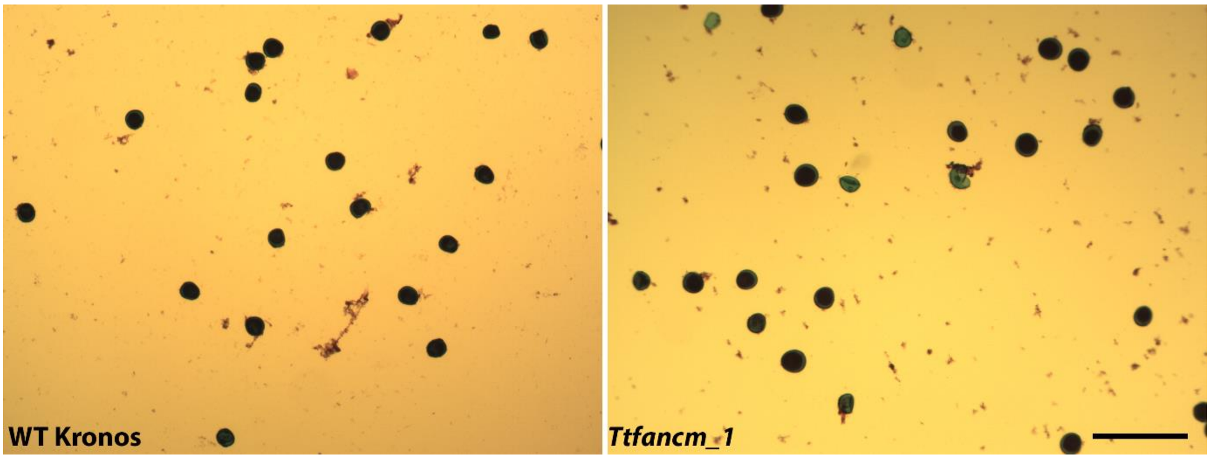

Supplementary Figure 2. Reduced pollen viability in *Ttfancm_1* null mutant. Alexander staining of fresh pollen grains. Representative pollen grains are shown from replicates, wild type (*n* = 2042) and *Ttfancm_1* (*n* = 1994). Scale bar = 200 μm.

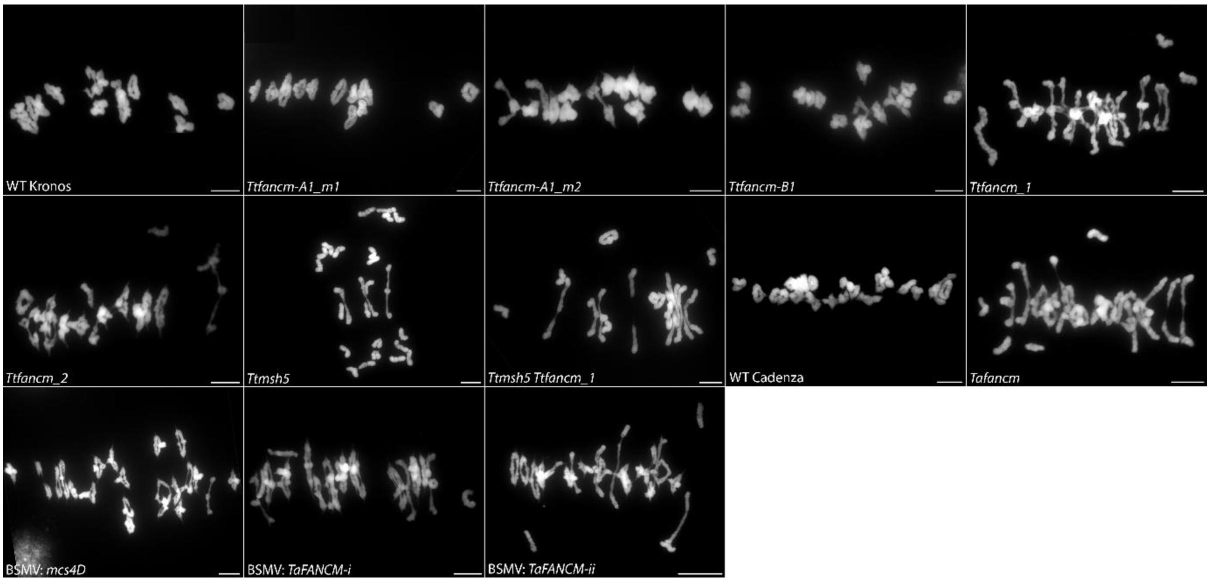

Supplementary Figure 3. FANCM promotes formation of obligate chiasma. DAPI-stained meiotic chromosome spreads at metaphase I. Representative micrographs are shown from replicates, WT Kronos (*n* = 60), *Ttfancm-A1_m1* (*n* = 64), *Ttfancm-A1_m2* (*n* = 60), *Ttfancm-B1* (*n* = 44), *Ttfancm_1* (*n* = 104), *Ttfancm_2* (*n* = 30), *Ttmsh5* (*n* = 194), *Ttmsh5 Ttfancm_1* (*n* = 86), WT Cadenza (*n* = 76), *Tafancm* (*n* = 81), BSMV: *msc4D* (*n* = 50), BSMV: *TaFANCM-i* (*n* = 33) and BSMV: *TaFANCM-ii* (*n* = 43). Scale bars = 10 µm.

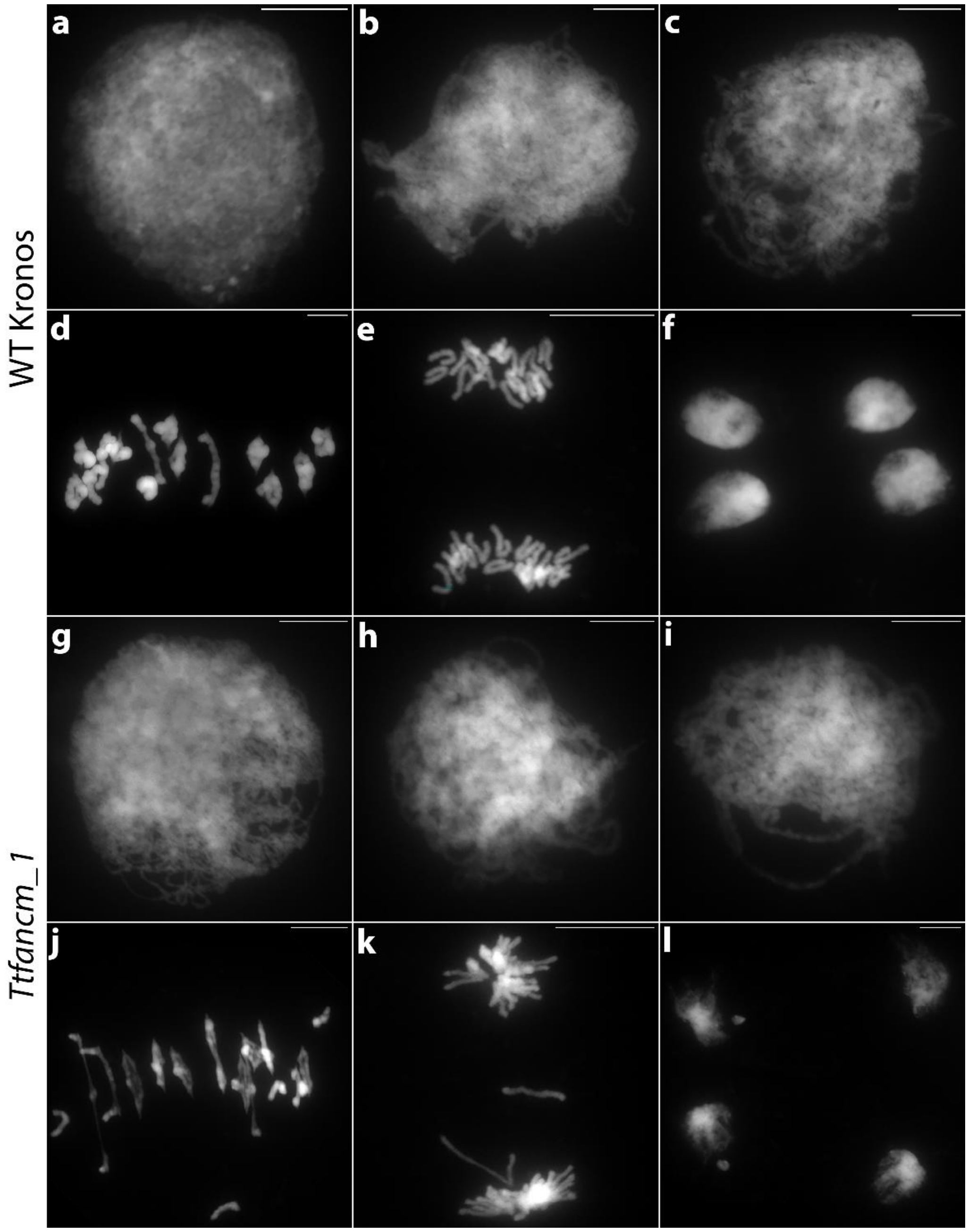

Supplementary Figure 4. Univalents and mis-segregation observed in *Ttfancm_1*, resulting in unbalanced gametes. a-l Meiotic atlas of DAPI-stained chromosome spreads. Representative micrographs are shown from replicates, **a** (*n* = 10), **b** (*n* = 10), **c** (*n* = 10), **d** (*n* = 60), **e** (*n* = 40), **f** (*n* = 10), **g** (*n* = 10), **h** (*n* = 10), **i** (*n* = 10), **j** (*n* = 60), **k** (*n* = 40), and **l** (*n* = 10). **a** and **g** Leptotene. **b** and **h** Zygotene. **c** and **i** Pachytene. **d** and **j** Metaphase I. **e** and **k** Dyad. **f** and **l** Tetrad. Scale bars = 10 µm.

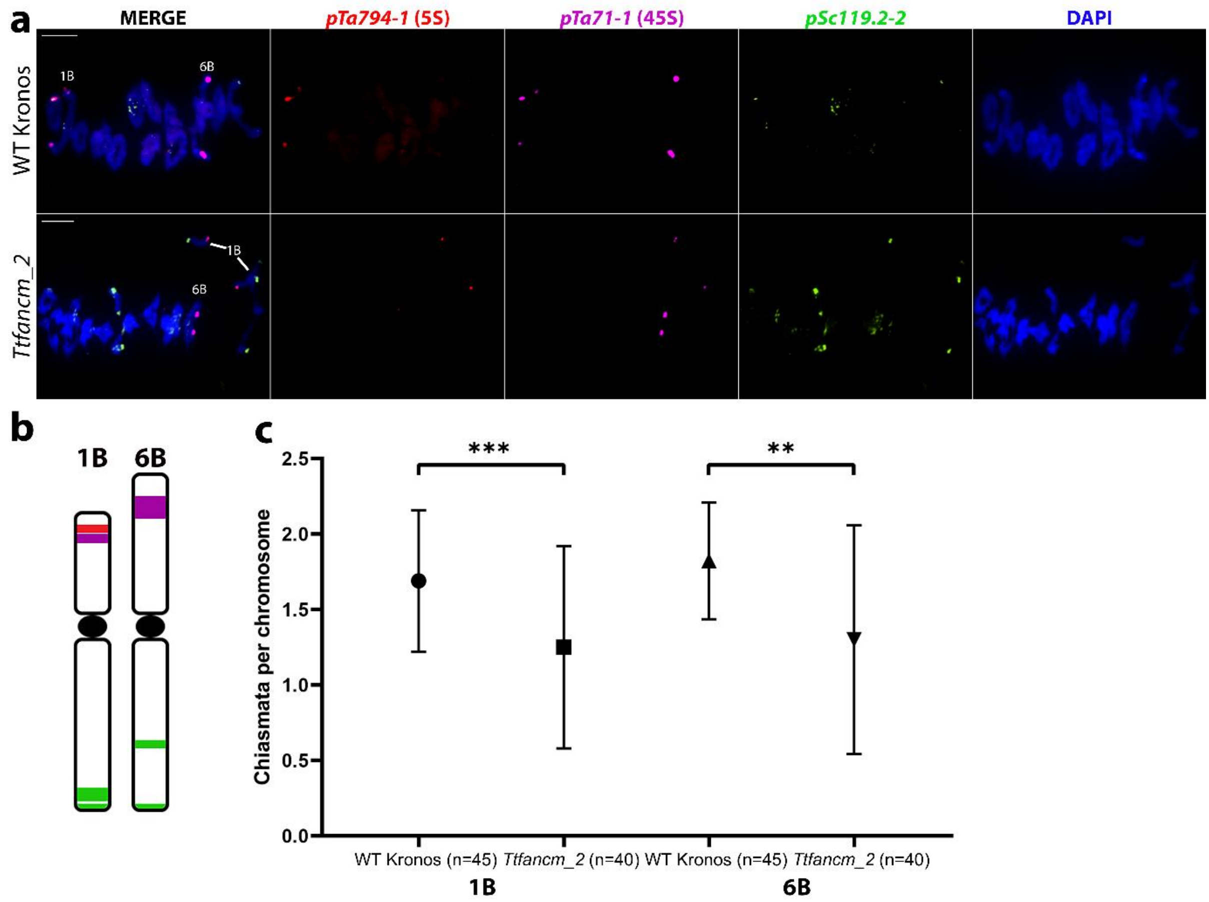

Supplementary Figure 5. Chiasmata number reduced in chromosomes 1B and 6B in *Ttfancm_2*. a Fluorescence *in situ* hybridization (FISH) of pTa794-1 (5*S*), pTa71-1 (45*S*) and pSc119.2-2 probes on meiotic metaphase I chromosome spreads. Representative micrographs are shown from replicates, WT Kronos (n = 45) and *Ttfancm_2* (n = 40). Scale bars = 10 µm. **b** Hybridization signal patterns of chromosomes 1B and 6B. **c** Mean ± SD chiasmata frequency per chromosome. The number of meiocytes sampled for each line is shown in brackets. ** = $p < 0.01$. *** = $p < 0.001$ (Mann-Whitney U Test). Exact p-values are as follows: 1 vs 2 (p = 0.002), 3 vs 4 ($p = 3.34 \times 10^{-4}$). Source data are provided as a Source Data file.

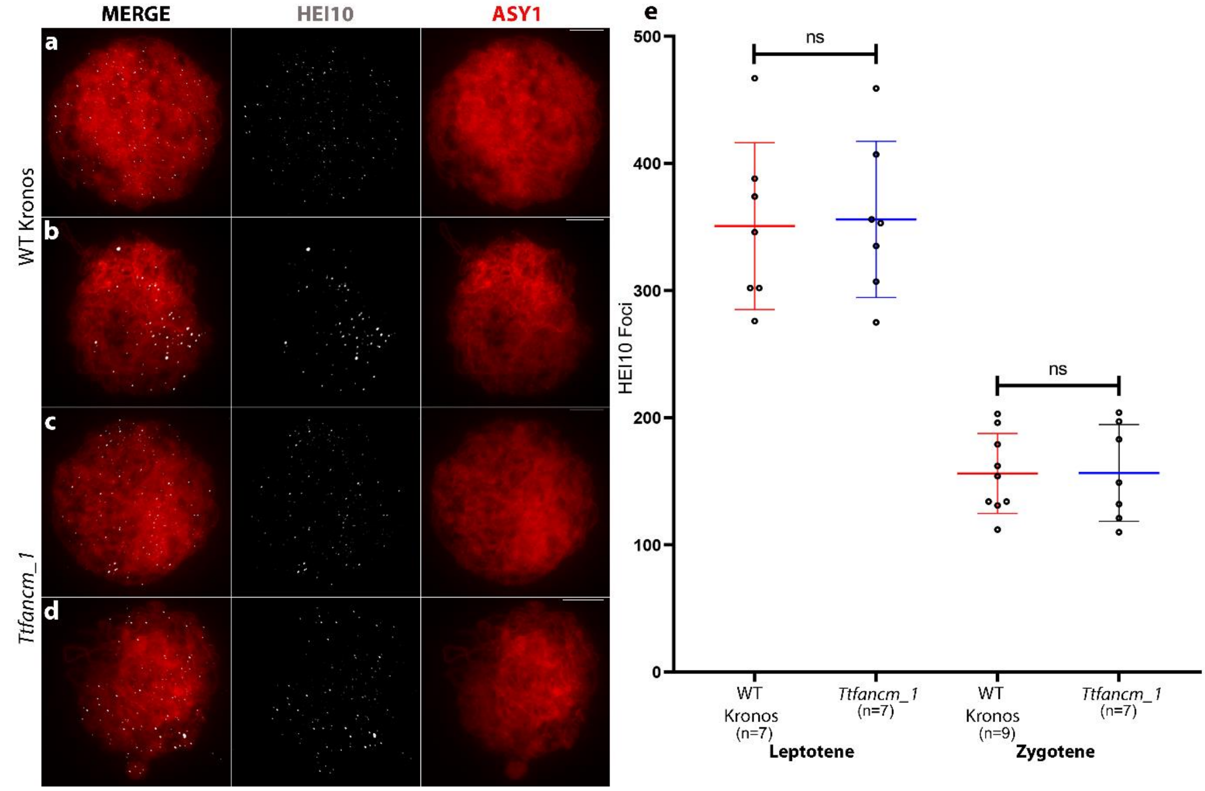

Supplementary Figure 6. Class I crossover recombination protein HEI10 is unaffected in the *Ttfancm_1* null mutant at early prophase I. a-e Co-immunofluorescence of HEI10 (white) and ASY1 (red) on meiotic prophase I chromosome spreads. Representative micrographs are shown from replicates, **a** (n = 7), **b** (n = 7), **c** (n = 9) and **d** (n = 7). Leptotene (**a** and **c**) and Zygotene (**b** and **d**). Scale bars = 10 µm. **e** Counts of HEI10 foci per cell with mean values ± SD. The number of cells sampled for each line is shown in brackets. n.s. = $p > 0.05$ (Mann-Whitney U Test). Exact p-values are as follows: 1 vs 2 (p = 1), 3 vs 4 (p = 1). Source data are provided as a Source Data file.

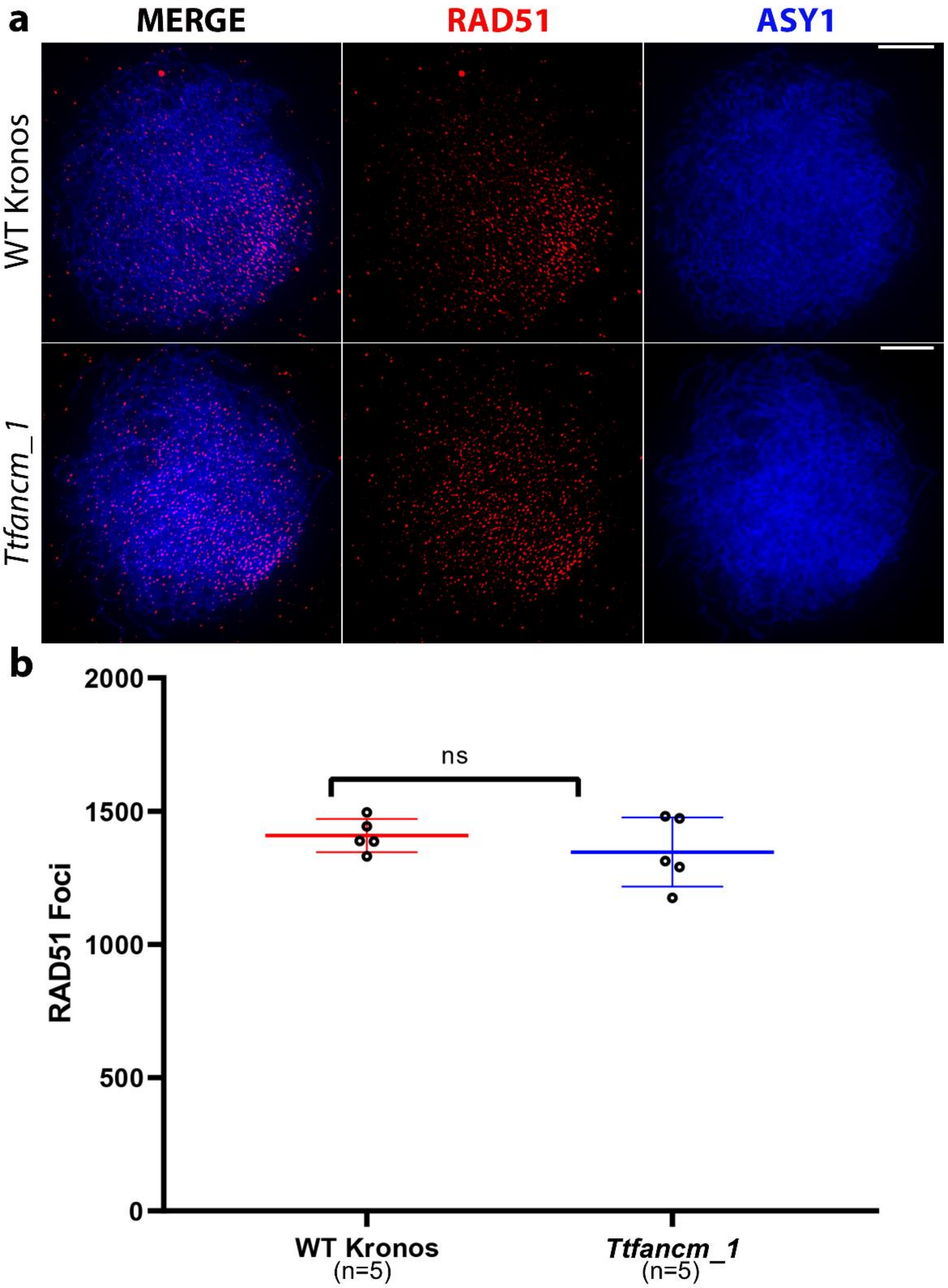

Supplementary Figure 7. Early recombination protein RAD51 is unaffected in the *Ttfancm_1* null mutant at leptotene. a Co-immunofluorescence of RAD51 (red) and ASY1 (blue) on meiotic chromosome spreads at leptotene. Representative micrographs are shown from replicates, WT Kronos (n = 5) and *Ttfancm_1* (n = 5). Scale bars = 10 µm. **b** Counts of RAD51 foci per cell with mean values ± SD. The number of cells sampled for each line is shown in brackets. n.s. = $p > 0.05$ (Mann-Whitney U Test). Exact p-value is $p = 0.403$. Source data are provided as a Source Data file.

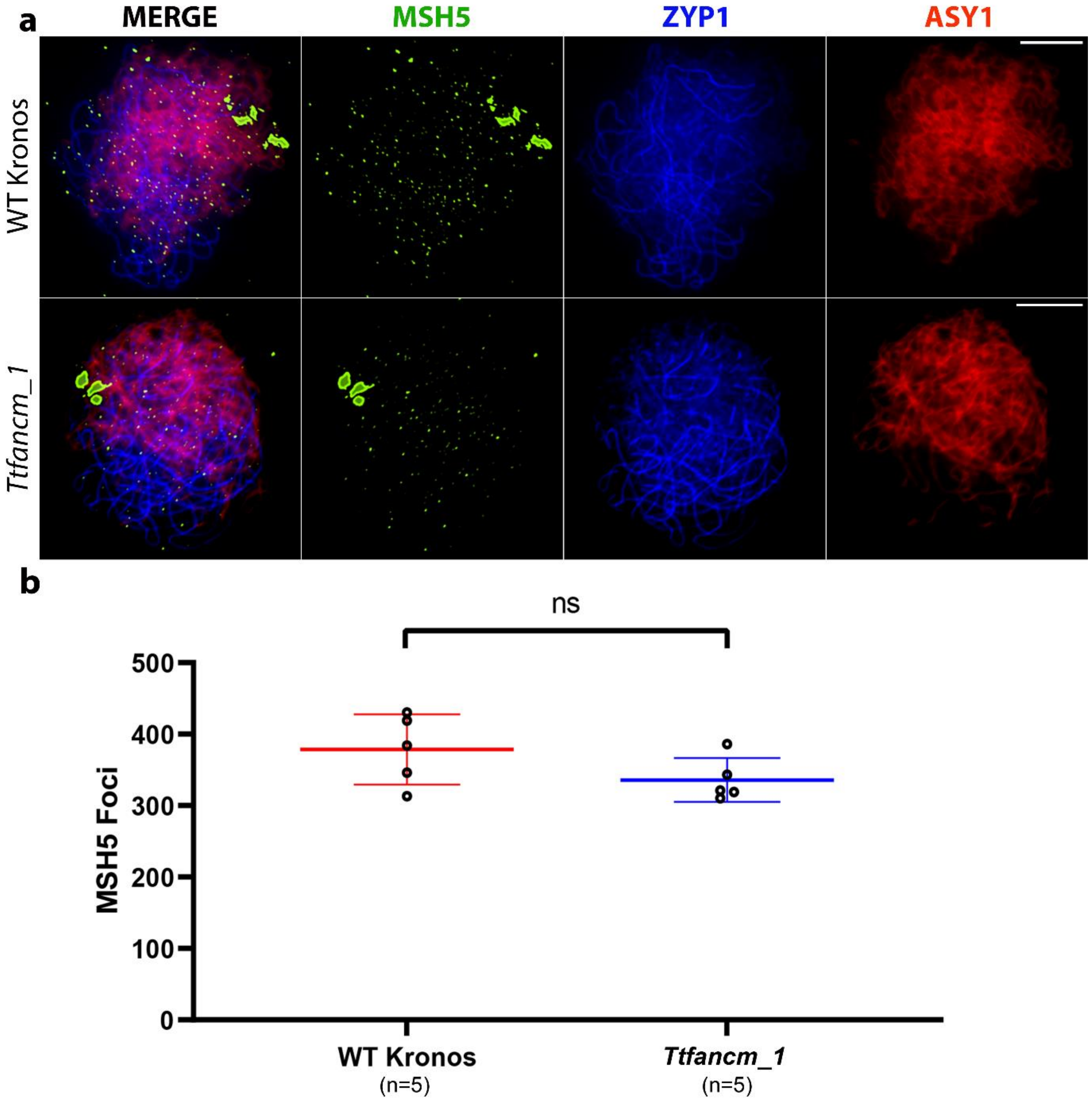

Supplementary Figure 8. Class I crossover recombination protein MSH5 is unaffected in the *Ttfancm_1* null mutant at mid-zygotene. a Co-immunofluorescence of MSH5 (green), ZYP1 (blue) and ASY1 (red) on meiotic chromosome spreads at mid-zygotene. Representative micrographs are shown from replicates, WT Kronos ($n = 5$) and *Ttfancm_1* ($n = 5$). Scale bars = 10 µm. **b** Counts of MSH5 foci per cell with mean values ± SD. The number of cells sampled for each line is shown in brackets. n.s. = $p > 0.05$ (Mann-Whitney U Test). Exact *p*-values is $p = 0.066$. Source data are provided as a Source Data file.

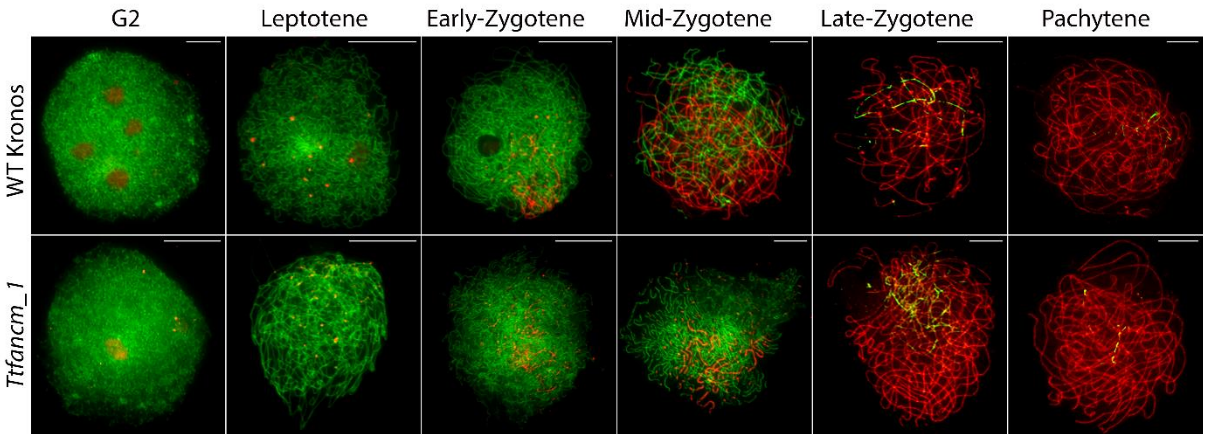

Supplementary Figure 9. Axis formation and synapsis are unaffected in the *Ttfancm_1* null mutant. Co-immunofluorescence of ASY1 (green) and ZYP1 (red) on meiotic prophase I chromosome spreads. Representative micrographs are shown from replicates: for WT Kronos G2 (*n* = 5), leptotene (*n* = 53), early-zygotene (*n* = 18), mid-zygotene (*n* = 6), late-zygotene (*n* = 9), pachytene (*n* = 11); for *Ttfancm_1* G2 (*n* = 5), leptotene (*n* = 20), early-zygotene (*n* = 17), mid-zygotene (*n* = 21), late-zygotene (*n* = 3), pachytene (*n* = 16). Scale bars = 10 µm.

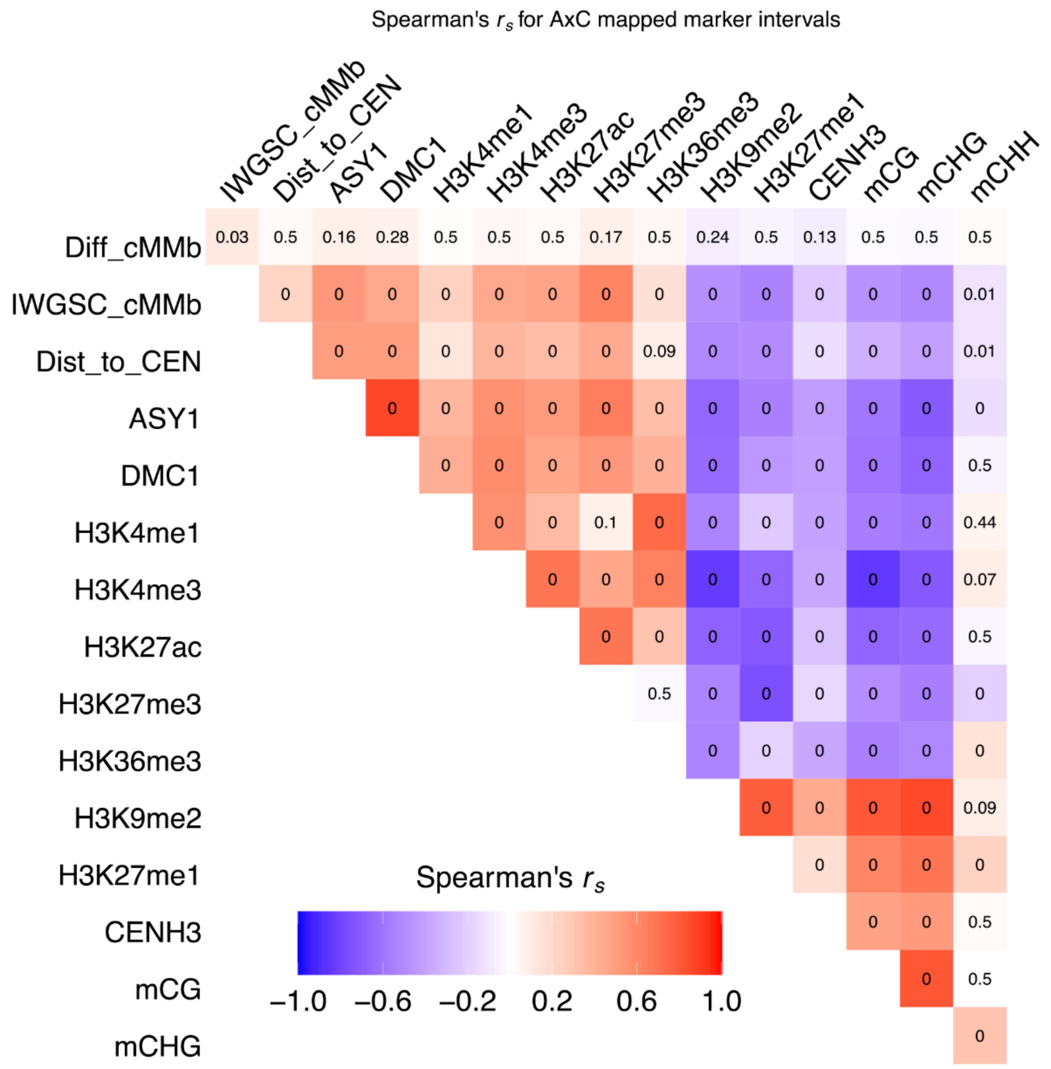

Supplementary Figure 10. Spearman's rank-order correlation coefficients (r_s) for the indicated parameter pairs computed within each genetic marker interval. Correlation coefficients are indicated by cell colour. *P*-values for r_s correlation coefficients were standardised to represent those based on pairwise values across 100 marker intervals and are indicated within each cell. Included data sets are differential CO rate (*fancm* cM/Mb minus wild type cM/Mb; "Diff_cMMb"), wild type CO rate derived from a Chinese Spring x Renan genetic map ("IWGSC_cMMb"), ASY1, DMC1, H3K4me3, H3K9me2 and H3K27me1 ChIP-seq, H3K4me1 and H3K27ac ChIP-seq, H3K27me3 and H3K36me3 ChIP-seq, CENH3 ChIP-seq, whole-genome bisulfite sequencing-derived DNA methylation (mCG, mCHH and mCHG proportions), and the distance between the midpoint of each marker interval and the midpoint of previously defined centromeric coordinates ("Dist_to_CEN").
