# Supplementary material for: FANCM promotes class I interfering crossovers and suppresses class II non-interfering crossovers in wheat meiosis
Source: Nat Commun. 2022 Jun 25;13:3644. doi: 10.1038/s41467-022-31438-6 (PMC9233680; doi:10.1038/s41467-022-31438-6)
Supplement: Supplementary file 3 — Description of Additional Supplementary Files [file 41467_2022_31438_MOESM3_ESM.pdf]

Description of Additional Supplementary Files

File Name: Supplementary Data 1
Description: TILLING mutants selected for characterisation of FANCM in tetraploid (cv. Kronos) and hexaploid (cv. Cadenza) wheat. Mutant ID is based on nomenclature used in the main text, whereas VariantID is the nomenclature used in EnsemblPlants based on Krasileva *et al*. 2017.

File Name: Supplementary Data 2
Description: Primer sequences (excluding tails) used to screen for mutations, germplasm development, and to screen the Kronos F3 populations. Note that these are KASP primers which is a three primer reaction including a common primer and two tailed primers for either the wild type (WT) or the EMS mutant allele.

File Name: Supplementary Data 3
Description: FANCM promotes formation of the obligate chiasma. Data expressed as mean values ± S.E.

File Name: Supplementary Data 4
Description: Complete dataset of Table 1 including details of Avalon x Cadenza F3 fancm mutant marker recombination analysis

File Name: Supplementary Data 5
Description: Complete dataset of Table 1 including details of Avalon x Cadenza F3 fancm mutant marker recombination analysis

File Name: Supplementary Data 6
Description: Complete dataset of Table 1 including details of Avalon x Cadenza F3 fancm mutant marker recombination analysis

File Name: Supplementary Data 7
Description: Complete dataset of Table 2 including details of Kronos F3 marker recombination analysis

File Name: Supplementary Data 8
Description: Differential crossover rate (fancm cM/Mb minus wild type cM/Mb), wild type crossover rate, and chromatin and meiotic protein mean signals, profiled via ChIP-seq or bisulfite-seq

File Name: Supplementary Data 9
Description: Summary data of model on differential crossover rate (fancm cM/Mb minus wild type cM/Mb), wild type crossover rate, and chromatin and meiotic protein mean signals, profiled via ChIP-seq or bisulfite-seq
