# Supplementary material for: FANCM promotes class I interfering crossovers and suppresses class II non-interfering crossovers in wheat meiosis
Source: Nat Commun. 2022 Jun 25;13:3644. doi: 10.1038/s41467-022-31438-6 (PMC9233680; doi:10.1038/s41467-022-31438-6)
Supplement: Supplementary file 13 — Reporting Summary [file 41467_2022_31438_MOESM13_ESM.pdf]

nature portfolio

Corresponding author(s): James Higgins

Last updated by author(s): 2022/06/14

Reporting Summary

Nature Portfolio wishes to improve the reproducibility of the work that we publish. This form provides structure for consistency and transparency in reporting. For further information on Nature Portfolio policies, see our Editorial Policies and the Editorial Policy Checklist.

Statistics

For all statistical analyses, confirm that the following items are present in the figure legend, table legend, main text, or Methods section.

n/a	Confirmed	
☐	☒	The exact sample size (*n*) for each experimental group/condition, given as a discrete number and unit of measurement
☒	☐	A statement on whether measurements were taken from distinct samples or whether the same sample was measured repeatedly
☐	☒	The statistical test(s) used AND whether they are one- or two-sided<br>*Only common tests should be described solely by name; describe more complex techniques in the Methods section.*
☒	☐	A description of all covariates tested
☐	☒	A description of any assumptions or corrections, such as tests of normality and adjustment for multiple comparisons
☐	☒	A full description of the statistical parameters including central tendency (e.g. means) or other basic estimates (e.g. regression coefficient) AND variation (e.g. standard deviation) or associated estimates of uncertainty (e.g. confidence intervals)
☐	☒	For null hypothesis testing, the test statistic (e.g. *F*, *t*, *r*) with confidence intervals, effect sizes, degrees of freedom and *P* value noted<br>*Give P values as exact values whenever suitable.*
☒	☐	For Bayesian analysis, information on the choice of priors and Markov chain Monte Carlo settings
☒	☐	For hierarchical and complex designs, identification of the appropriate level for tests and full reporting of outcomes
☒	☐	Estimates of effect sizes (e.g. Cohen's *d*, Pearson's *r*), indicating how they were calculated

Our web collection on statistics for biologists contains articles on many of the points above.

Software and code

Policy information about availability of computer code

Data collection	GeneTitan using standard affymetrix protocols was used for Cadenza recombination map analysis. Axiom Analysis Suite (version 3.1.51.0) was used to assign genotype calls. NIS elements (Nikon) was used to capture and process images.
Data analysis	Minitab v 18 was used for statistical analysis. Genetic mapping was carried out for each region individually using MSTmap online (http://www.mstmap.org/). Spearman's rank-order correlation coefficients (rs) were computed for each parameter pair and plotted as a correlation matrix, with rs denoted by cell colour (R v 4.0.0).

For manuscripts utilizing custom algorithms or software that are central to the research but not yet described in published literature, software must be made available to editors and reviewers. We strongly encourage code deposition in a community repository (e.g. GitHub). See the Nature Portfolio guidelines for submitting code & software for further information.

Data

Policy information about availability of data

All manuscripts must include a data availability statement. This statement should provide the following information, where applicable:

- Accession codes, unique identifiers, or web links for publicly available datasets
- A description of any restrictions on data availability
- For clinical datasets or third party data, please ensure that the statement adheres to our policy

Provide your data availability statement here.

Human research participants

Policy information about studies involving human research participants and Sex and Gender in Research.

Reporting on sex and gender	n/a
Population characteristics	n/a
Recruitment	n/a
Ethics oversight	n/a

Note that full information on the approval of the study protocol must also be provided in the manuscript.

Field-specific reporting

Please select the one below that is the best fit for your research. If you are not sure, read the appropriate sections before making your selection.

[x] Life sciences [ ] Behavioural & social sciences [ ] Ecological, evolutionary & environmental sciences

For a reference copy of the document with all sections, see nature.com/documents/nr-reporting-summary-flat.pdf

Life sciences study design

All studies must disclose on these points even when the disclosure is negative.

Sample size	Sample size was not pre-calculated but determined when enough data points reliably fitted a statistical model. For example, we have previously shown that class II COs fit a Poisson distribution in Desjardins et al. 2020. However, if sample size is less than 100 then the randomness of the phenomena means that the model fits less well. The sample size depends on the number of chromosomes in wheat. In species with fewer chromosomes, such as Arabidopsis, only 50 samples are needed to fit the distribution. We are then able to standardize our sampling size based on these numbers.
Data exclusions	there were no data exclusions except monomorphic markers which are uninformative for the analysis.
Replication	replication is shown by the n = in the main body of text.
Randomization	samples were not randomized as all elements were counted in the experiments. In the recombination mapping experiments, we analysed all plants and in the cytological analysis we captured all meiotic nuclei on the slides. The anthers used to make the slides were from the primary wheat spikes for consistency of samples between plants. Plants were genotyped, and anthers collected.
Blinding	Blinding was not possible because capturing and scoring the meiotic cells requires a specific skill set that could only be performed by the first author. Blinding could not be performed with the recombination mapping either.

Reporting for specific materials, systems and methods

We require information from authors about some types of materials, experimental systems and methods used in many studies. Here, indicate whether each material, system or method listed is relevant to your study. If you are not sure if a list item applies to your research, read the appropriate section before selecting a response.

Materials & experimental systems

n/a	Involved in the study
[ ]	[x] Antibodies
[ ]	[ ] Eukaryotic cell lines
[ ]	[ ] Palaeontology and archaeology
[ ]	[ ] Animals and other organisms
[ ]	[ ] Clinical data
[ ]	[ ] Dual use research of concern

Methods

n/a	Involved in the study
[ ]	[ ] ChIP-seq
[ ]	[ ] Flow cytometry
[ ]	[ ] MRI-based neuroimaging

Antibodies

Antibodies used	Primers AbF 5’-AGCATATGTTGGTGGCAGCTGGTGTG-3’ and AbR 5’- TCCTCGAGATTGTATTCCCCAGCTCG-3’ were used to amplify 1107-1935 bp of the TraesCS4B02G096400 (FANCM-B1) coding region with Q5 DNA polymerase (NEB). The PCR product was ligated

	into pDrive (Qiagen) and sequenced (Eurofins). pDrive was then digested with NdeI and XhoI, and the insert was ligated into pET21b (Merck-Millipore). Transformed BL21 (DE3) cells expressed a 32 kDa recombinant protein that was used to produce a rat anti-FANCM antibody (DC Biosciences).<br>Immunostaining was conducted using the following primary antibodies: anti-TaASY1 guinea pig, 1:500 (Armstrong et al 2002); anti-anti-AtZYP1C rat, 1:500 (Higgins et al. 2005); anti-AtZYP1C rabbit, 1:500 (Osman et al 2018); anti-AtRAD51 rabbit, 1:200 (Mercier et al 2003); anti-MSH5 rabbit 1:200 (Higgins et al 2008); anti-HvHEI10 rabbit, 1:250 (Desjardins et al 2020). Secondary antibodies that were used, 1:200: goat anti-guinea pig AMCA (Jackson ImmunoResearch); goat anti-guinea pig Alexa Fluor® 488 (Abcam); goat anti-guinea pig Alexa Fluor® 594 (Abcam); goat anti-rat AMCA (Jackson ImmunoResearch); goat anti-rat Alexa Fluor® 594 (Invitrogen); goat anti-rabbit AMCA (Jackson ImmunoResearch); goat anti-rabbit Alexa Fluor® 488 (Invitrogen) and goat anti-rabbit DyLight® 594 (Vector Labs).
Validation	anti-wheat fancm rat was validated against the null fancm mutant presented in figure 4. Validation of the other antibodies is presented in the references cited above in the antibodies used section or on company websites.

Eukaryotic cell lines

Policy information about cell lines and Sex and Gender in Research

Cell line source(s)	n/a
Authentication	n/a
Mycoplasma contamination	n/a
Commonly misidentified lines (See ICLAC register)	n/a

Palaeontology and Archaeology

Specimen provenance	n/a
Specimen deposition	n/a
Dating methods	n/a

☐ Tick this box to confirm that the raw and calibrated dates are available in the paper or in Supplementary Information.

Ethics oversight	n/a

Note that full information on the approval of the study protocol must also be provided in the manuscript.

Animals and other research organisms

Policy information about studies involving animals; ARRIVE guidelines recommended for reporting animal research, and Sex and Gender in Research

Laboratory animals	n/a
Wild animals	n/a
Reporting on sex	n/a
Field-collected samples	n/a
Ethics oversight	n/a

Note that full information on the approval of the study protocol must also be provided in the manuscript.

Clinical data

Policy information about clinical studies

All manuscripts should comply with the ICMJEguidelines for publication of clinical research and a completedCONSORT checklist must be included with all submissions.

Clinical trial registration	n/a
Study protocol	n/a
Data collection	n/a
Outcomes	n/a

Dual use research of concern

Policy information about dual use research of concern

Hazards

Could the accidental, deliberate or reckless misuse of agents or technologies generated in the work, or the application of information presented in the manuscript, pose a threat to:

No	Yes	
x		Public health
x		National security
x		Crops and/or livestock
x		Ecosystems
x		Any other significant area

Experiments of concern

Does the work involve any of these experiments of concern:

No	Yes	
x		Demonstrate how to render a vaccine ineffective
x		Confer resistance to therapeutically useful antibiotics or antiviral agents
x		Enhance the virulence of a pathogen or render a nonpathogen virulent
x		Increase transmissibility of a pathogen
x		Alter the host range of a pathogen
x		Enable evasion of diagnostic/detection modalities
x		Enable the weaponization of a biological agent or toxin
x		Any other potentially harmful combination of experiments and agents

ChIP-seq

Data deposition

- [ ] Confirm that both raw and final processed data have been deposited in a public database such as GEO.
- [ ] Confirm that you have deposited or provided access to graph files (e.g. BED files) for the called peaks.

Data access links *May remain private before publication.*	n/a
Files in database submission	n/a
Genome browser session (e.g. UCSC)	n/a

Methodology

Replicates	n/a
Sequencing depth	n/a
Antibodies	n/a
Peak calling parameters	n/a
Data quality	n/a
Software	n/a

Flow Cytometry

Plots

Confirm that:

- [ ] The axis labels state the marker and fluorochrome used (e.g. CD4-FITC).
- [ ] The axis scales are clearly visible. Include numbers along axes only for bottom left plot of group (a 'group' is an analysis of identical markers).
- [ ] All plots are contour plots with outliers or pseudocolor plots.
- [ ] A numerical value for number of cells or percentage (with statistics) is provided.

Methodology

Sample preparation	n/a
Instrument	n/a
Software	n/a
Cell population abundance	n/a
Gating strategy	n/a

- [ ] Tick this box to confirm that a figure exemplifying the gating strategy is provided in the Supplementary Information.

Magnetic resonance imaging

Experimental design

Design type	n/a
Design specifications	n/a
Behavioral performance measures	n/a

Acquisition

Imaging type(s)	n/a
Field strength	n/a
Sequence & imaging parameters	n/a
Area of acquisition	n/a

Diffusion MRI [ ] Used [ ] Not used

Preprocessing

Preprocessing software	n/a
Normalization	n/a
Normalization template	n/a
Noise and artifact removal	n/a
Volume censoring	n/a

Statistical modeling & inference

Model type and settings	n/a
Effect(s) tested	n/a

Specify type of analysis: [ ] Whole brain [ ] ROI-based [ ] Both

Statistic type for inference (See Eklund et al. 2016)	n/a

Correction	n/a

Models & analysis

n/a	Involved in the study
☒	☐ Functional and/or effective connectivity
☒	☐ Graph analysis
☒	☐ Multivariate modeling or predictive analysis
